# Artificial intelligence‐assisted ultrasound‐guided regional anaesthesia: An explorative scoping review

**DOI:** 10.1002/jeo2.12104

**Published:** 2024-08-14

**Authors:** Martina Marino, Rebecca Hagh, Eric Hamrin Senorski, Umile Giuseppe Longo, Jacob F. Oeding, Bengt Nellgard, Anita Szell, Kristian Samuelsson

**Affiliations:** ^1^ Fondazione Policlinico Universitario Campus Bio‐Medico Via Alvaro del Portillo Roma Italy; ^2^ Research Unit of Orthopaedic and Trauma Surgery, Department of Medicine and Surgery Università Campus Bio‐Medico di Roma, Via Alvaro del Portillo Roma Italy; ^3^ Sahlgrenska Sports Medicine Center Gothenburg Sweden; ^4^ Department of Health and Rehabilitation, Institute of Neuroscience and Physiology, Sahlgrenska Academy University of Gothenburg Gothenburg Sweden; ^5^ Department of Orthopaedics, Institute of Clinical Sciences, Sahlgrenska Academy University of Gothenburg Gothenburg Sweden; ^6^ School of Medicine Mayo Clinic Alix School of Medicine Rochester Minnesota USA; ^7^ Department of Anesthesiology and Intensive Care, Institute of Clinical Sciences, Sahlgrenska Academy University of Gothenburg Gothenburg Sweden

**Keywords:** convolutional neural networks, landmark identification, machine learning algorithms, tracking algorithms

## Abstract

**Purpose:**

The present study reviews the available scientific literature on artificial intelligence (AI)‐assisted ultrasound‐guided regional anaesthesia (UGRA) and evaluates the reported intraprocedural parameters and postprocedural outcomes.

**Methods:**

A literature search was performed on 19 September 2023, using the Medline, EMBASE, CINAHL, Cochrane Library and Google Scholar databases by experts in electronic searching. All study designs were considered with no restrictions regarding patient characteristics or cohort size. Outcomes assessed included the accuracy of AI‐model tracking, success at the first attempt, differences in outcomes between AI‐assisted and unassisted UGRA, operator feedback and case‐report data.

**Results:**

A joint adaptive median binary pattern (JAMBP) has been applied to improve the tracking procedure, while a particle filter (PF) is involved in feature extraction. JAMBP combined with PF was most accurate on all images for landmark identification, with accuracy scores of 0.83, 0.93 and 0.93 on original, preprocessed and filtered images, respectively. Evaluation of first‐attempt success of spinal needle insertion revealed first‐attempt success in most patients. When comparing AI application versus UGRA alone, a significant statistical difference (*p* < 0.05) was found for correct block view, correct structure identification and decrease in mean injection time, needle track adjustments and bone encounters in favour of having AI assistance. Assessment of operator feedback revealed that expert and nonexpert operator feedback was overall positive.

**Conclusion:**

AI appears promising to enhance UGRA as well as to positively influence operator training. AI application of UGRA may improve the identification of anatomical structures and provide guidance for needle placement, reducing the risk of complications and improving patient outcomes.

**Level of Evidence:**

Level IV.

**Abbreviations**
AI: artificial intelligence
C‐COT: continuous convolution operator's tracker
CNT: convolutional network‐based tracker
DCNNs: deep convolutional neural networks
ECO: efficient convolution operators
FT‐AMBP: adaptive median binary pattern
HDT: hedged deep tracking
HOG: histograms of oriented gradients
JAMBP: joint adaptive median binary pattern
KLT: Kanade–Lucas–Tomasi
LoE: level of evidence
ML: machine learning
MLAs: machine learning algorithms
MS: mean shift
PF: particle filter
SANet: structure‐aware network
UGRA: ultrasound‐guided regional anaesthesia
VAS: visual analogue scale

## INTRODUCTION

Over the last two decades, ultrasound‐guided regional anaesthesia (UGRA) has become the gold standard for regional nerve blocks [12]. The procedure provides advantages in direct visualization of structures including neurovascular bundles, muscle, tendon, and bone, and facilitates visualization of injectate spread, in addition to enabling a reduced dosage of local anaesthetic administration, leading to an overall improvement of block procedure and success [12, 30].

Despite its many benefits, UGRA is complicated by several technical challenges, especially rampant among trainee physicians [3, 30]. Physicians may experience loss of reflective signal between the needle and the probe leading to decreased needle visibility in overweight patients or during deep blocks, and hyperechoic structures may also lead to impairing needle visibility [30]. The presence of speckle noise and artefacts is common, and image interpretation can be limited by these features and may, as a result, heavily rely on the level of expertise of the anaesthesiologist [13, 29]. Beyond operator experience, image interpretation by humans is limited by a variety of parameters including structure noise, incomplete visual search patterns, fatigue, distractions, vast amounts of image data and image quality [13].

The introduction of artificial intelligence (AI) applications to a variety of medical fields, including various radiological imaging tasks, has revealed the potential of resolving procedural faults in UGRA. This can be done by providing AI systems that are able to identify landmarks guiding operators to more accurate block procedures [13]. Furthermore, AI‐guided solutions have the potential to improve the interpretation of the sonographic image and the visualization of needle advancement and local anaesthetic injection [30].

These AI systems usually take computer‐extracted (radiomic) features that serve as input to machine learning algorithms (MLAs) that will subsequently ‘learn’ a task to execute given the specific input data provided [13]. Deep learning is a subcategory of machine learning (ML) in which multiple‐layered networks are used to assess complex patterns within the raw imaging input data; this may be conducted via deep convolutional neural networks (DCNNs) [13]. DCNNs' design is inspired by the biological processes occurring in the visual cortex of animals and they are well‐suited for analysing visual imagery; thus, they have the strength for more complex classification problems with automatic feature extraction [30]. In general, the application of these ML approaches, in which multiple radiomic features are merged into a single value, in the case of UGRA are usually aimed at producing feature extracting (landmark identification) and feature tracking (needle tracking) algorithms [13, 30].

The application of such AI models to UGRA is promising and is expected to increase during the 2020s. On the one hand, the use of AI has the potential to provide assistance for novice trainees and experienced clinicians, possibly improving first‐attempt success and procedural accuracy and reducing procedural time [30]. On the other hand, there may still be incidents of tracking failures or errors and there remains limited evidence available on the accuracy of AI‐assisted UGRA in many different patient populations [30]. The present study serves as an explorative scoping review on the topic of AI‐assisted UGRA and discusses the related intraprocedural parameters, postprocedural outcomes and operator feedback reported in the literature.

## METHODS

### Protocol and registration

A formal review protocol was not created.

### Eligibility criteria

Considering the authors' proficiency in various languages, studies in English, Italian and Swedish were screened. Included studies had to report investigations on AI‐assisted UGRA. AI assistance was considered to be application of any MLA, DCNN, Feature Tracking or Extracting Algorithm or any other commercial AI application or program. Peer‐reviewed studies of each level of evidence according to Oxford classification were included. Technical notes, letters to editors and conference commentaries were considered for inclusion. All study designs were considered with no restrictions regarding patient characteristics or cohort size. Instructional courses or studies including procedures other than UGRA were excluded. In vitro, animal, cadaver and biomechanical studies were not considered.

### Information sources

A review of literature was performed using the Preferred Reporting Items for Systematic reviews and Meta‐Analyses extension for Scoping Reviews (PRISMA‐ScR) guidelines [6]. A systematic electronic literature search was conducted on September 19th 2023 using the Medline, EMBASE, CINAHL, Cochrane Library and Google Scholar databases by an expert in electronic searching.

### Search

The initial search strategy was organized according to the PICO (Population, Intervention, Comparison, Outcome) structure. Patients undergoing UGRA (P) assisted by AI (I) were included in the review. The intraprocedural and postprocedural parameters of UGRA with or without AI assistance were compared (C). Intraprocedural parameters and postprocedural outcomes were extracted (O).

The search strategies used a combination of Medical Subject Heading (MeSH) terms and a ‘title/abstract’ search. For all databases, a similar search strategy to the following was used: ‘(ultrasound OR ultrasonography OR ultrasonics) AND (regional anaesthesia OR conduction anaesthesia OR local anaesthesia OR nerve block) AND (artificial intelligence OR AI OR machine learning OR ML OR deep learning)’. Differences in search keys were due to database configuration. All search details can be seen in Appendix 1.

### Selection of sources of evidence

**Figure 1:**
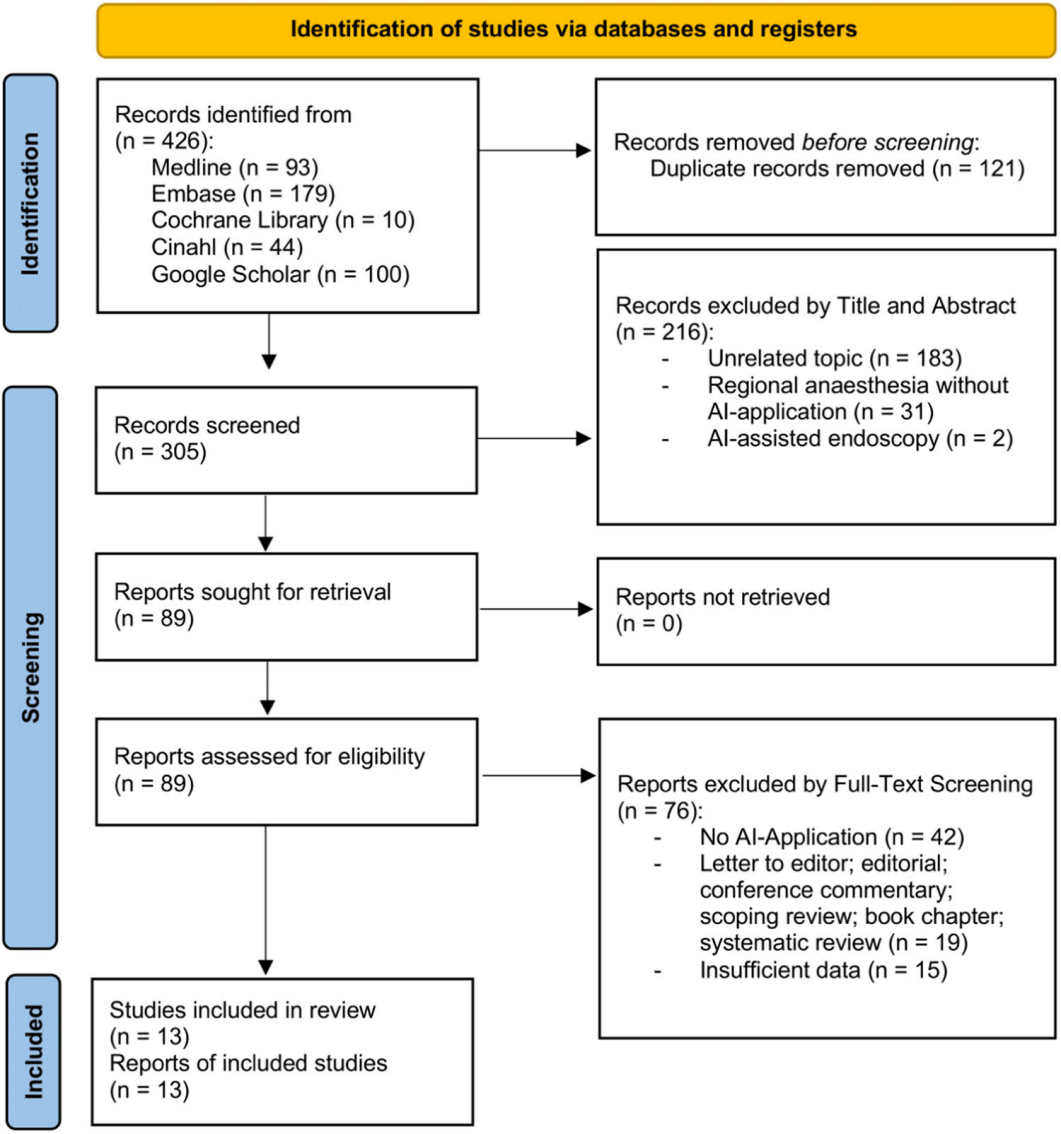
PRISMA flow chart. PRISMA 2020 flow diagram for new systematic reviews which included searches of databases and registers only. From: Page et al. [25]. For more information, visit: http://www.prisma-statement.org/.

Study screening was performed by two independent reviewers (Martina Marino and Rebecca Hagh) starting with title and abstract screening followed by full‐text evaluation. Data extraction was performed by the same reviewers. Differences, at any stage, were reconciled by mutual agreement and in case of disagreement, a third reviewer was consulted for consensus (Kristian Samuelsson). Guidelines by Moher et al. were followed to design the PRISMA chart (Figure 1) [23].

### Data charting process

Draft data charting tables for recording extracted data from the included articles were created using Microsoft Excel (Version 16.16.1[22101101]) based on the scoping review research statement formulated using the PICO approach. Data charting was piloted by two reviewers (Martina Marino and Rebecca Hagh) and uncertainties or discrepancies in the process were discussed and resolved via consultation with the other team members.

### Data items

General study characteristics extracted include author; year of publication; type of study; level of evidence (LoE); intended function of AI model; number of participants; and specific AI model utilized.

Data were charted into predetermined tables, categorized based on the parameters being assessed: the first reports on the accuracy of AI‐model tracking (Table 1), the second evaluates success at the first attempt with the application of MLA (Table 2), the third reports on differences in outcomes between AI‐assisted and unassisted UGRA (Table S1), the fourth table reports operator feedback on AI‐assisted UGRA (Table S2); and finally, the last (fifth) table displays case reports on the use of AI‐assisted regional anaesthesia for peripheral nerve blocks (Table 3).

**Table 1:** Accuracy of artificial intelligence (AI) model tracking.

Reference	Type of study, level of evidence (LoE)	Function	Participants	AI model	Accuracy (%)							Timing (s)
					Image type			Nerve				
					Original	Preprocessed	Filtered	Median	Sciatic	Overall		
Alkhatib et al. [2]	Prospective case‐series study, iV	Peripheral nerve identification from US images	10	**Feature tracking algorithm + Feature extracting algorithm** PF + JAMBP PF + AMBP PF + MBP PF + LBP PF + CLBP PF + JCLBP PF + Hist PF + HOG PF + Gabor	0.89* 0.86 0.78 0.76 0.78 0.80 0.62 0.74 0.54	0.93* 0.88 0.82 0.81 0.82 0.84 0.72 0.75 0.59	0.93* 0.90 0.84 0.83 0.85 0.88 0.72 0.82 0.58					
				MS + JAMBP MS + AMBP MS + MBP MS + LBP MS + CLBP MS + JCLBP MS + Hist MS + HOG MS + Gabor	0.78* 0.74 0.53 0.56 0.56 0.71 0.48 0.50 0.47	0.89* 0.80 0.78 0.78 0.79 0.83 0.74 0.60 0.52	0.91* 0.88 0.83 0.82 0.84 0.89 0.76 0.68 0.54					
				KLT + JAMBP KLT + AMBP KLT + MBP KLT + LBP KLT + CLBP KLT + JCLBP KLT + Hist KLT + HOG KLT + Gabor	0.65 0.51 0.47 0.42 0.41 0.59 0.43 0.66* 0.64	0.78 0.71 0.69 0.60 0.63 0.70 0.40 0.79* 0.76	0.81* 0.70 0.71 0.62 0.63 0.73 0.41 0.80 0.76					
Alkhatib et al. [1]	Prospective case‐series study, iV	Peripheral nerve identification from US images	42	**Tracking algorithms** C‐COT ECO CNT MDNet SANet SiameFC CFNet DCFNet MCPF HDT HCFT CREST DLT FT‐AMBP				0.94* 0.94* 0.79 0.93 0.94* 0.82 0.85 0.86 0.89 0.87 0.85 0.92 0.86 0.87	0.73 0.80* 0.73 0.73 0.76 0.74 0.77 0.73 0.79 0.80* 0.78 0.73 0.75 0.71	0.84 0.87* 0.76 0.82 0.85 0.78 0.81 0.79 0.84 0.83 0.82 0.83 0.80 0.82		
Hetherington et al. [16]	Prospective case‐series study, iV	Identification of vertebral level from US images	20	**CNN** AlexNet GoogLeNet ResNet‐50 SqueezeNet							88.7 ± 9.1 88.4 ± 8.3 90.8 ± 6.8 87.8 ± 8.4	0.343 ± 10 0.488 ± 14 0.416 ± 6 0.44 ± 2
Ikhsan et al. [17]	Prospective case‐series study, IV	Spinal landmark identification from US images (L3/4 interspinous space) with the addition of the Gabor Filter	40	MLA + Gabor Filter							100	30.9
Leng et al. [20]	Prospective case‐series study, IV	Spinal landmark identification from US images (L3/4 interspinous space)	Previous Spinal Disorder History: 8 No History: 45	MLA							87.5 95.6	146 90

*Note*: * Indicates the highest score.

Abbreviations: C‐COT, continuous convolution operators tracker; CFNet, correlation filter network; CNN, convolutional neural network; CNT, convolutional network based tracker; CREST, convolutional residual tracker; DCFNet, discriminant correlation filters network; DLT, deep‐learning tracker; ECO, efficient convolution operators; FT‐AMBP, particle filter adaptive median binary pattern; HCFT, hierarchical convolutional features tracker; HDT, hedged deep tracking; LoE, level of evidence; MCPF, multitask correlation particle filter; MDNet, multidomain convolutional neural networks; SANet, structure‐aware network; SiameFC, fully convolutional Siamese networks; US, ultrasound.

**Table 2:** Success at first attempt with application of machine learning algorithm (MLA).

Reference	Type of study, level of evidence (LoE)	Function	Participants	Artificial intelligence (AI) model	Success of spinal needle insertion		Average number of puncture attempts	Average number of attempts to identify L3/L4	Program recorded depth of the skin to posterior complex and the clinician measured depth correlation	
					First attempt	Not First attempt				
									Pearson's	Cronbach's
In Chan et al. [18]	Prospective cohort study, II	Spinal landmark identification from ultrasound (US) images (L3/4 interspinous space)	48 (obese)	MLA (trained using historical data collected from previous studies of research group)	38	10 (6 = second) (2 = third) (2 = fourth)	1.3 (SD = 0.75)		0.915	0.956
Oh et al. [24]	Prospective cohort study, II	Spinal landmark identification from US images (L3/4 interspinous space)	100	MLA‐Intelligent image processing system (training based on anatomical landmark images from patient's database)	92	8 (5 = second) (3 = third)		3.1 (SD = 3.0)	0.94	0.97

**Table 3:** Case reports on the use of artificial intelligence (AI)‐assisted regional anaesthesia for peripheral nerve blocks.

Reference	Type of study, level of evidence (LoE)	Function	Participant *n*°	AI model	Patient demographics					Block site	Regions identified by AI model	Time to complete block procedure (mins)	Drug and volume administered	Postoperative VAS				
					Sex	Age	Height (cm)	Weight (kg)	ASA status					Hour 0	Hour 2	Hour 6	Hour 12	Hour 24
Erdem et al. [10]	Case report, IV	Landmark and nerve identification from US images	1	(CNN) Nerveblox	Male	42	184	93	II	Infraclavicular block	Pectoralis major muscle; Pectoralis minor muscle; Subclavian artery; Subclavian vein	4	20 cc of 5% bupivacaine	0	0	0	0	2
			2		Female	48	165	95	II			5		0	0	0	0	0
			3		Female	34	172	88	II	Pectoral nerve (PECS) block	Pectoralis major muscle; Pectoralis minor muscle; Serratus anterior muscle; Parietal pleura; First rib		10 cc of 0.25% bupivacaine	0	0	0	0	3

Abbreviations: ASA, American Society of Anaesthesiologists physical status; VAS, visual analogue scale.

### Critical appraisal of individual sources of evidence

**Figure 2:**
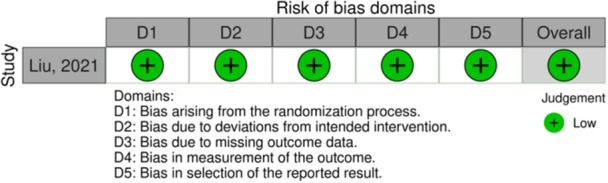
RoB2: Risk of bias assessment for randomized controlled trials.

**Figure 3:**
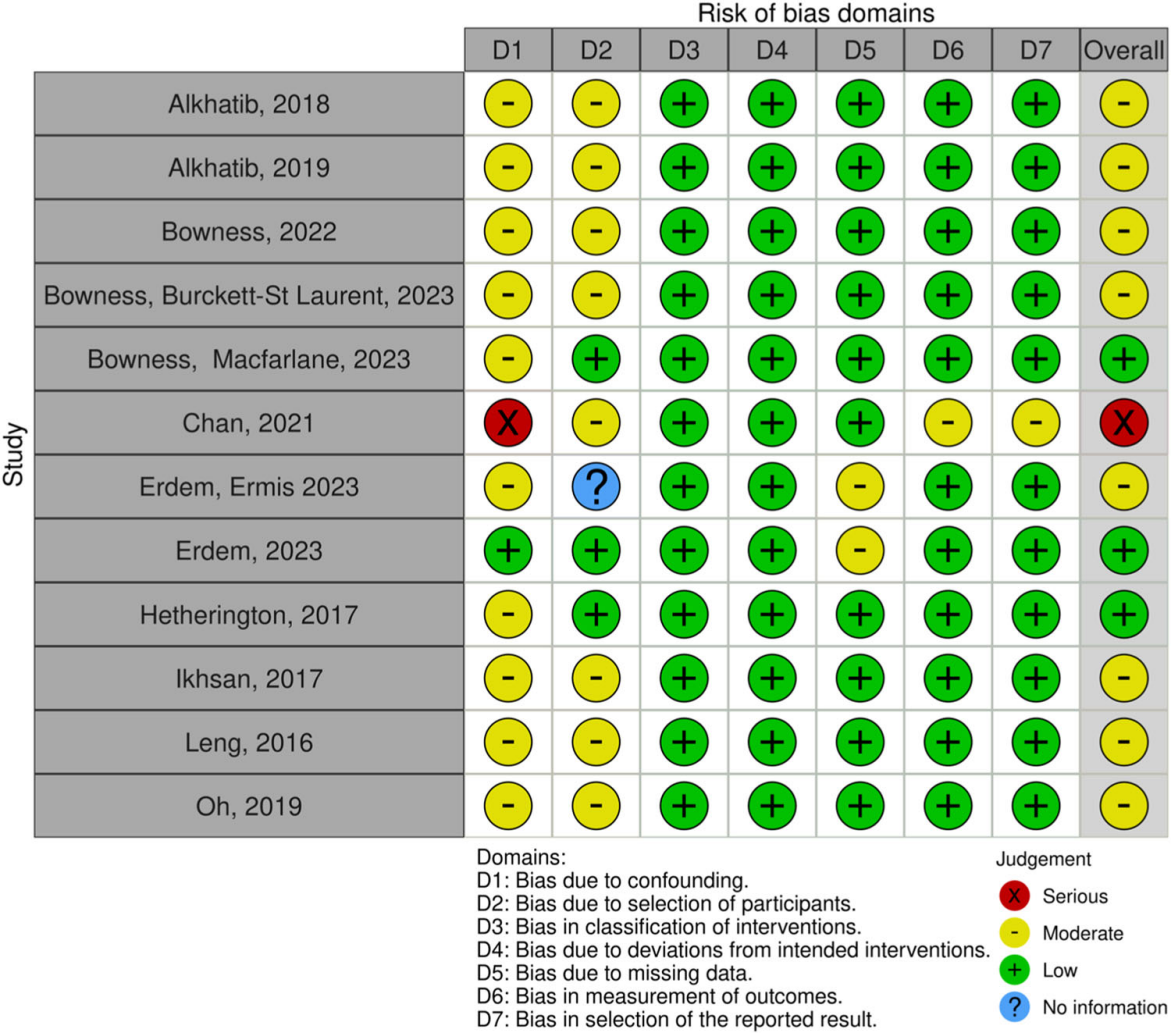
ROBINS‐I: Risk of bias in nonrandomized studies of interventions.

Given the designs of the included studies, the Risk of Bias (RoB 2) tool for Randomized Trials [28] (Figure 2) and the Risk Of Bias In Non‐randomised Studies of Interventions (ROBINS‐I) [27] were used for quality assessment (Figure 3).

The Rob2 and ROBINS‐I tools both define a set of domains and provide ‘signalling questions’ designed to evaluate different biases within a study. The former is applied exclusively to randomized trials while the latter may be applied to non‐randomized studies of interventions. Each domain can be scored as having a low, moderate, serious or critical risk of bias, and an overall score can be assigned to each study.

Risk of bias evaluation in the context of the present review is useful for an overall assessment of the quality of the current available literature on the topic, and for highlighting specific concerns that should be kept in consideration for future investigations. Additionally, it provides a measure of the reliability of the reported studies' findings.

### Synthesis of results

No statistical analysis of the results was carried out, charted data were reported in the results section according to the table which they belonged (Tables 1, 2, 3).

Given the heterogeneity of reported results, tables were created based on the outcomes assessed in each study, allowing for better grouping and comparison of similar results. In light of this, studies were placed into five groups including those assessing the accuracy of AI‐model tracking; those reporting first‐attempt success as the primary outcome; those comparing AI‐assisted and unassisted UGRA; and finally, case reports detailing intraprocedural and postprocedural parameters. Once studies were grouped, the five tables were named and set up according to the reported data.

## RESULTS

### Selection of sources of evidence

The literature search identified 426 studies from databases. Duplicate removal resulted in the exclusion of 121 studies. Two‐hundred and sixteen studies were excluded during title and abstract screening, reasons for exclusion were studies discussing an unrelated topic (183); UGRA without application of AI (31); and AI‐assisted endoscopy (2). Seventy‐six studies were excluded during full‐text screening. Forty‐two did not discuss AI application, 19 were formulated as editorial commentary or book chapters and the rest had insufficient data (15). Thus, 13 studies met the selection criteria and were included in the present review. The flowchart of the literature search is reported in Figure 1.

### Characteristics of sources of evidence

The LoE of each of the included studies was one level I randomized control trial; [21] three level II cross‐sectional studies; [5, 7, 10] two level II prospective cohort studies; [18, 24] one level III prospective interventional study; [6] five level IV prospective case‐series studies; [1, 2, 16, 17, 20] one level IV case report [11].

The 13 studies reviewed included a total of 461 participants and each study reported on feature extracting algorithms; feature tracking algorithms; MLA; DCCNs algorithms and/or specific AI programs applied to UGRA. All algorithms were applied for landmark and/or nerve identification. In some cases, AI was applied to recorded patient data sets that included US images or videos of UGRA procedures [1, 2, 16]. In others, participants were either recruited for US scanning only [5, 6, 7, 17, 20] or for perioperative AI‐assisted UGRA procedures [10, 11, 18, 22, 24].

Reported outcomes included the accuracy of AI‐model tracking [1, 2, 16], first attempt success [18, 24], operator feedback [5, 6, 7, 10, 22] and procedural outcomes of individual case reports [11].

### Critical appraisal within sources of evidence

The included RCT [22] was judged as having a ‘low risk of bias’ for each of the five evaluated domains (Figure 2).

When applying the ROBINS‐I risk of bias tool (Figure 3), studies with only one domain classified as having a moderate risk of bias were considered as having an overall low risk of bias [6, 11, 16]. Those with two or more domains classified as moderate risk were considered as having an overall moderate risk of bias [1, 2, 5, 7, 10, 17, 20, 24]. If studies were evaluated as having one or more domains classified as serious risk, they were considered as having an overall serious risk of bias [18].

Only one study was found to have a low risk of confounding [11]. In some cases, confounding variables went unmeasured especially due to random, unspecified or non‐stratification of specific patient characteristics in relation to their possible effects on the outcomes of the intervention [1, 2, 16, 18, 24]. Additionally, in some cases, the risk of confounding bias was attributed to the fact that measures for distinguishing between expert and non‐expert participants were vague, and thus could influence results [5, 6, 7, 10].

Eight studies had a moderate risk of bias for the selection of participants [1, 2, 5, 6, 7, 17, 20, 24] and one did not state any selection criteria at all [10]. Selection criteria for participants were overall not clearly stated and often participant characteristics were not reported nor were results stratified accordingly.

Two studies had a moderate risk of bias due to missing data, one was due to loss to follow‐up [10] and the other because there was data that were not reported in one patient compared to the rest [11].

One study [18], considered as having an overall serious risk of bias, was found to have a moderate risk of bias in the measurement of outcomes and the selection of reported results. This was because authors used different modes available on the specific AI algorithm, an ‘obese mode’ and ‘normal mode’; however, results were not stratified based on which was applied.

### Results of individual sources of evidence

Note that Appendix 2 displays a list of the most relevant algorithms and models discussed and reported in the included studies, along with a brief description of their characteristics and whether they are commercial or open source.

#### Accuracy of AI‐model tracking (Table 1)

Five of the included studies reported on the accuracy of AI‐model tracking [1, 2, 16, 17, 20] and results are reported in Table 1. All tracking algorithms were tested on ultrasound (US) images, and in all studies accuracy of structure identification was measured out of 1. There was moderate evidence for bias due to confounding in all five studies, while four had moderate risk for participant selection, suggesting that possible confounding factors were not accounted for, and that participant selection should be more transparently reported in the future.

##### Feature tracking algorithms plus feature extracting algorithms

One study [2] reported on a combination of feature tracking algorithms (particle filter [PF]; mean shift [MS]; Kanade–Lucas–Tomasi [KLT]) with feature extracting algorithms for peripheral nerve identification. Accuracy was further stratified based on application to original, preprocessed and filtered images.

Joint adaptive median binary pattern (JAMBP) combined with PF had the highest accuracy scoring 0.83, 0.93 and 0.93 on original, preprocessed and filtered images, respectively. The lowest accuracy was reported by combining PF with Gabor filter accuracy: 0.54 for original, 0.59 for preprocessed and 0.58 for filtered images. All other accuracy scores ranged between 0.62 and 0.90.

JAMBP combined with MS scored highest on accuracy values (0.79 for original, 0.89 for preprocessed, 0.91 for filtered); however, accuracy was lower overall when compared to the application of JAMBP with PF. The lowest accuracy was reported when combining MS with Gabor (0.47 for original, 0.52 for preprocessed, 0.54 for filtered) and values were also overall lower when compared to Gabor applied to PF.

The highest accuracy values were found when combining KLT with Histograms of Oriented Gradients (HOG) for original (0.66) and preprocessed (0.79) images. While KLT and JAMBP combined scored highest on filtered images (0.81). Overall KLT combinations performed worst in terms of accuracy compared to PF and MS.

##### Tracking algorithms for peripheral nerve identification

One study [1] evaluated specific tracking algorithms for peripheral nerve identification and stratified for median and sciatic nerves in addition to providing an overall accuracy score.

For median nerve identification, the most successful tracking algorithms were continuous convolution operator's tracker (C‐COT), efficient convolution operators (ECO) and structure‐aware network (SANet) all of which scored 0.94. The least accurate algorithm, convolutional network‐based tracker (CNT), scored 0.79 for median nerve identification.

Accuracy for sciatic nerve identification was lower overall compared to that obtained for the median nerve. The highest reported accuracy value was 0.80 thanks to the application of ECO and hedged deep tracking (HDT). The lowest accuracy, 0.79, was recorded when using particle filter adaptive median binary pattern (FT‐AMBP).

The highest value for combined accuracy was reported to be 0.87 thanks to the application of ECO. The lowest value for combined identification accuracy was 0.76 when using CNT.

##### Accuracy of AI‐model for vertebral level identification

One study [16] evaluated vertebral level identification accuracy. The most accurate CNN was ResNet‐50 (0.908 ± 6.8), but it was also the second slowest (416 ± 6 ms). SqueezeNet scored lowest on accuracy (87.8 ± 8.4) but was fastest (44 ± 2 ms).

Another study [17] evaluated the accuracy of a spinal identification MLA software combined with a Gabor filter on 40 patients and found 100% accuracy with an average scanning time of 30.9 s.

Finally, the last study [20] evaluating accuracy using an MLA software without filter application on a total of 53 volunteers found an accuracy of 87.5% and 95.6% on patients with previous history of spinal disorders and those without, respectively. Scanning time took an average of 146 s in those eight patients with a history of spinal disorder, and 90 in the 45 with no history.

#### Success at first attempt with application of machine learning algorithm (MLA) (Table 2)

The two studies [18, 24] in Table 2 report on the first attempt success of spinal needle insertion with the application of MLA designed for landmark identification from US images. Of the included studies, Chan et al. [18]. were considered as having a serious overall risk of bias due to moderate risk in participant selection and serious risk for unaccounted confounding variables, especially related to the selection of obese participants. The second study was evaluated as having an overall moderate risk of bias due to moderate risk of confounding and for participant selection.

One study [18] included 48 obese patients and in 38 cases spinal needle insertion was successfully carried out at the first attempt. Average number of puncture attempts was 1.3 (SD = 0.75). The program recorded the depth of the skin to posterior complex and the clinician measured depth correlation via Pearson's and Cronbach's correlations, which were reported to be 0.915 and 0.956, respectively. This study was classified as having a serious risk of bias overall due to the lack of stratification of results based on the application of ‘obese mode’ or ‘normal mode’ when utilizing the MLA. Additionally, BMI was used for classifying pregnant women as obese without discussing how or if they adjusted the measurement based on this parameter.

One study [24] included 100 patients, 92 of which had successful spinal needle insertion at the first attempt. Pearson's and Cronbach's correlations were reported to be 0.94 and 0.97, respectively. Finally, the authors reported an average of 3.1 (SD = 3.0) attempts before L3/L4 vertebral identification.

#### Differences in outcomes between AI‐assisted and unassisted UGRA (Table S1)

Both studies [6, 22] included in Table S1 evaluate differences in outcomes based on AI‐assisted or unassisted landmark and nerve identification from US images. Both studies had an overall low risk of bias.

One study [6] evaluated landmark and nerve identification and reports on a variety of outcomes. ‘ScanNav’ AI model was used, and 21 non‐experts were enroled for experimentation on three participants. Significant statistical differences (*p* < 0.05) in outcomes were found for the identification of correct block view and correct structure identification in favour of having AI assistance.

One study [22] evaluated peripheral nerve identification by 100 operators and reported on numerous outcomes when applying a CNN model known as ‘SegNet’. A statistically significant difference (*p* < 0.05) in favour of AI application was found for decreased mean injection time, decreased needle track adjustments and decreased number of times bone was encountered. AI application also yielded better results in terms of reported complications. Thus, only one patient reported complications in this group of patients as opposed to seven in the control group.

#### Operator feedback (Table S2)

Table S2 includes three studies [5, 7, 10] that reported on feedback following the application of AI to UGRA specifically for nerve and landmark identification. All three studies scored as having a moderate risk of bias due to the potential of confounding and in participant selection. In one case, there was no information at all on the selected participants, which raises concerns regarding study quality.

One study [5] stratified feedback based on expert or nonexpert operators. Thus, 240 scans were performed, of which 120 were carried out with the application of ‘ScanNav’, while the rest of the patients served as the control group. Most non‐expert operators had a positive response to ‘identifying structures’, ‘learning scan’, ‘helped with training’, and ‘confidence’ when AI was applied (51.7%, 60%, 61.7% and 51.7%, respectively). The majority of participants had a neutral response to ‘acquisition of correct view’ and ‘supervisor support’ (61.7% and 85%, respectively). Non‐experts provided positive feedback more frequently and provided negative feedback less frequently than experts (*p* = 0.001). Non‐expert median confidence in their own scanning was 6 (interquartile range [IQR] 5–8) without ScanNav and 7 (IQR 5.75–9) with ScanNav (*p* = 0.07). Thus, 50% of the experts reported positive responses to AI applications for teaching and 48.3% reported increased confidence in supervising non‐experts. Furthermore, most experts reported neutral responses regarding frequency of intervention (56.7%), and confidence in own scanning (73.3%). Similarly, 45% responded positively and 48.3% responded neutrally for application to supervising.

One study [7] assessed ScanNav and reported on the perceived accuracy assessment of peripheral nerve block, and the influence of highlighting on the risk of adverse events and block failure. Total accuracy (combined true positive and true negative identification) when using the program was found to be 0.935. Totally, 62.9% of experts agreed that there was a reduced likelihood of nerve injury/postoperative neurological symptom; 86.2% reported reduced local anaesthetic and systemic toxicity; 76.2% reported reduced events of pneumothorax; 82.5% agreed that there was a reduced likelihood of peritoneal penetration; and finally, 81.2% reported reduced likelihood of block failure.

One study [10] utilized ‘Nerveblox’, where 40 operators were asked about their experience when applying this program. Results demonstrated that 87.5% of operators agreed that its application would be more effective in deep nerve blocks; it will enhance block success; it will reduce the number of block attempts; and will accelerate block area localization. Furthermore, 80% agreed that it would increase patient comfort and that it contribute to developing new blocks and techniques. Also, 72.5%, and 82.5%, agree, respectively, that it will decrease the required drug volume and concentration and that it will be suitable for routine use. Moreover, 95% agree that possible complications will be reduced. Finally, 90% of experts agreed that it will facilitate self‐learning and 100% agreed that it will facilitate learning of regional block. Additionally, statistically significant differences (*p* < 0.001) were found in time reduction when using AI assistance to perform infraclavicular and pectoralis nerve blocks.

#### Case‐report postprocedural outcomes (Table 3)

One study reported on the application of ‘Nerveblox’ on landmark and nerve identification using US in three patients. The risk of bias was overall low. Two patients underwent infraclavicular block, with procedural time being 4 min in the first patient and 5 min in the second. The first patient reported a postoperative visual analogue scale (VAS) of 0 at hours 0, 2, 6 and 12, and then reported a score of 2 at 24 hours. The second patient sustained a score of 0 up to 24 h. The third patient underwent pectoral nerve block, procedural time was not reported, and the VAS score was 0 at 0, 2, 6 and 12 h but reached 3 at 24 hours. Results are reported in Table 3.

## DISCUSSION

The application of AI to UGRA brings promising technological advancement. The scientific literature on AI‐assisted UGRA highlights the possibility of increased accuracy for landmark identification, high rates of first‐attempt success for spinal anaesthesia, better procedural outcomes in some cases compared to traditional UGRA and finally positive expert and non‐expert operator feedback. These outcomes are positive and demonstrate the early successes of AI‐assisted UGRA procedures. However, the risk of bias evaluation revealed concerns for unaccounted confounding variables and inadequate participant selection. This suggests that future studies, presumable randomized, should consider the possibility and attempt to mitigate the presence of confounding variables and perform more targeted participant selection. Additionally, heterogeneity in reporting calls for more targeted, high‐quality studies, that define, and report standardized outcomes of AI application to UGRA.

The use of AI shows promising results in the field of US imaging, particularly in the identification of anatomical landmarks. Results suggest that regardless of the kind of feature tracking and feature extracting algorithm used, accuracy tends to be high for landmark identification, although some combinations, such as those utilizing the JAMBP algorithm [2, 30], are especially accurate in making such predictions. JAMBP has been applied in various studies to improve the tracking procedure and automatic detection of nerve structures and regions of interest [2, 16, 30]. It has been found to enhance the accuracy and efficiency of UGRA by providing better real‐time identification of important anatomical structures [2, 16, 30]. This may not only increase the success rate of these procedures, but it may also reduce potential complications. In accordance with such findings, a study conducted on the use of AI for real‐time landmark identification in US‐guided thoracic paravertebral block demonstrated high accuracy for both paravertebral space and lung and bone identification [31]. Another study, assessing the accuracy of an AI‐based real‐time anatomy identification software for US‐guided peripheral nerve block procedures, also reported successful interpretation of anatomical structures [14]. These findings suggest that AI can significantly enhance the accuracy of landmark identification in US images, potentially improving clinical outcomes and patient care. Overall, it seems like feature tracking and feature extracting algorithms, alone and/or in combination, hold significant potential for advancing the field of US‐guided medical procedures. However, more research is needed to further validate programs’ individual effectiveness, accuracy and applicability in different clinical scenarios.

To study the level of clinical success of AI application to the UGRA procedure, some studies reported tangible outcomes including first‐attempt success rate for spinal anaesthesia and postoperative VAS score [18, 24]. Two studies applied a MLA, trained using historical data, to spinal anaesthesia administration, and in both cases, high first‐attempt success rates were reported [18, 24]. Furthermore, another study reporting on procedural outcomes in three patients undergoing infraclavicular and pectoral nerve blocks performed with CNN assistance (Nerveblox) reported on VAS values over 24 h [11]. These outcomes may be used in the assessment of the success of the block procedure and in both cases displayed promising results for AI application [11]. However, of the eleven included studies, none reported on standardized clinical outcomes that could be compared to evaluate the procedure. Finding and standardizing measures to evaluate patient safety, efficacy of the procedure, precision of drug dose and administration, incidence of adverse events and other parameters is crucial for a deeper and systematic understanding of the ramifications of AI application to UGRA and is currently missing from published studies.

Beyond the positive outcomes reported in unremarkable patient populations, some studies suggest that these algorithms can be designed and exploited for patients with specific characteristics that can lead to procedural difficulty [6, 18, 22]. One of the included studies [18] evaluated the possible applications of AI‐assisted UGRA to obese patients, representing a possible target population that may have characteristics leading to increased operational complexity. Administering spinal anaesthesia in obese patients presents several challenges [9, 26], the biggest of which may be difficulty in identifying anatomical landmarks due to increased adipose tissue, which can compromise correct needle placement [8, 26]. These factors may also lead to an increased risk of failed or inadequate spinal anaesthesia on the first attempt [8, 26]. AI application may improve the interpretation of sonographic images, visualization of needle advancement and injection of local anaesthetic in the context of altered landmark visibility [30]. These challenges reveal the necessity to test these programs according to specific populations to evaluate their success or failure, and the possibility of designing more targeted algorithms. Currently, there is little literature evaluating success in specific target populations, and available data, for example, that reported in the study by Chan et al., [18] would benefit from more transparent reporting of guidelines utilized for patient selection.

Another possible consideration with regard to AI‐assisted UGRA is how it performs against traditional procedures. Although results from studies comparing the application of AI, and lack thereof, are promising and suggest the possible procedural advantages including accuracy of placement of anaesthetic, efficiency in the operating room, enhanced patient comfort and satisfaction and potentially the reduction of risks associated with prolonged procedures (including nerve injury). Conclusively, statistically significant results comparing the two are not yet available [6, 19, 22]. ScanNav, for example, has shown promising results in three of the reviewed studies [5, 6, 7] and is an AI‐powered tool that utilizes MLA to assist in landmark identification by overlaying colour highlights to identify relevant anatomical structures in real‐time [5]. Over half of the questioned expert and nonexpert operators responded positively when asked about its possible usage for structure identification and helping in both training and teaching, the latter being another potential advantage of this technology [7]. However, definitive data on standardized outcomes evaluating the procedure are not available and are needed to provide definitive answers on whether their application provides better outcomes compared to standard procedure and to confirm that the benefits outweigh the costs of this technology.

Beyond successful clinical outcomes, the application of AI to UGRA may improve the procedure by providing higher‐quality education to trainees. UGRA poses a variety of challenges for trainees, including the presence of artefacts that affect image quality, difficulty in guiding the needle due to the necessity for high dexterity and high spatial orientation skills to achieve the correct manipulation and finally choosing the optimal technique and approach [4, 15]. AI programs may aid in learning and facilitating UGRA in several ways including in aiding in image interpretation by enhancing landmarks localization and increasing needle visibility, but it may also be applied to the development of simulations that can be used for the scope of training and making the learning process more efficient and safer [4, 15]. Overall, facilitation and increased quality of training have the potential to improve operator skills, ultimately leading to procedures that are carried out by more competent individuals and that may even lead to better outcomes. Thus, although this technology holds promise, operator feedback cannot be the only means of evaluation for the success of these algorithms in educational scenarios.

Currently, there seems to be a surplus of available algorithms for application to UGRA, that is, commercial models including ScanNav, NerveBlox and Segnet, while a variety of other algorithms and MLA systems were applied. Based on the current literature, it is crucial to evaluate, in a standardized manner, whether the application of such algorithms is indeed advantageous, and if such investigations lead to further improvement of the available systems, or the development of new algorithms that solve issues in previous versions. Currently, data are promising but are neither in‐depth nor standardized enough to provide solid, statistically significant information on the success of these models and for the development of protocols for their use. Further studies, on different populations, and for different purposes, should be designed while keeping in mind and accounting for the risk of bias for confounding (factors) and patient selection especially. Thus, it is important to begin and continue rigorous clinical trials, while also addressing ethical considerations, to ensure the responsible and beneficial application of this technology.

The present scoping review has several limitations, including the lack of statistical analysis. Unfortunately, due to the heterogeneity of the data available, it was not possible to perform a comparative meta‐analysis. Nevertheless, the inclusion and discussion of these data points provide an initial interpretation of the possible trajectory of AI‐assisted UGRA, and their interpretation is still worthwhile for the development of new studies investigating more standardized outcomes. Some of the included studies were judged to be of moderate or serious risk of bias overall. However, these were included regardless due to the small data available regarding the present topic. These studies still serve a purpose in the preliminary evaluation of possible applications of AI to UGRA. Finally, follow‐up in the included studies, for evaluation of postprocedural outcomes, was essentially immediate, excluding one study which reported on VAS at 24 h. This does not allow for consideration of possible complications reported by patients and future investigations should take this into account. Although the presence of a longer follow‐up may be beneficial, results evaluating immediate outcomes are also crucial given the nature of this procedure.

## CONCLUSIONS

AI appears promising to enhance UGRA as well as to positively influence operator training. AI application of UGRA can also help to improve the identification of anatomical structures and provide guidance for needle placement, reducing the risk of complications and may lead to improved patient outcomes. Despite moderate or serious risk of bias being attributed to many of the included studies, these still serve a purpose in the preliminary evaluation of possible applications of AI to UGRA. However, it highlights the need for more methodologically rigorous research in this field.

## AUTHOR CONTRIBUTIONS

**Martina Marino**: Methodology; formal analysis; investigation; data curation; writing—original draft preparation; visualization. **Rebecca Hagh**: Methodology; formal analysis. **Eric Hamrin Senorski**: Writing—review and editing. **Umile Giuseppe Longo**: Conceptualization; software; validation; writing—review and editing; supervision; project administration. **Jacob F. Oeding**: Writing—review and editing. **Bengt Nellgard**: writing—original draft preparation. **Anita Szell**: Writing—original draft preparation; writing—review and editing. **Kristian Samuelsson**: Conceptualization; writing—review and editing; supervision; project administration. All authors have read and agreed to the published version of the manuscript.

## CONFLICT OF INTEREST STATEMENT

The authors declare no conflict of interest.

## ETHICS STATEMENT

Not applicable.

## Supporting information

Supporting information

Supporting information

## Data Availability

All data generated or analysed during this study are included in this published article and its supplementary information files.

## APPENDIX 1

**Search strategy according to database**

**Table:**

	**Datum: 230921**
**Sökningen är beställd av:** Namn: Kristian Samuelsson Tfn: 0760405070 E‐post: kristian@samuelsson.cc Arbetsplats: Verksamhet ortopedi	**Sökningen är gjord av/search made by:** Namn: Ida Stadig Tfn: 031‐342 95 59 E‐post: ida.stadig@vgregion.se **Bibliotekariens arbetstid: 23 tim**

**Sökfråga/search question:** Does ultrasound‐guided regional anaesthesia with AI affect the time to apply the nerve block? Are there any differences in the effect of regional anaesthesia when using ultrasound‐guided regional anaesthesia with and without AI? Are there any differences regarding complications when using ultrasound‐guided regional anaesthesia with and without AI?

**Eventuella avgränsningar**

**Språk/language**: engelska, svenska, italienska

**Tid: ‐**

**Studietyp: ‐**

**Sökresultat: 305 referenser**

**Databaser/söktjänster som använts (databases used):** Medline (Ovid), Embase (Ovid), Cinahl, Cochrane Library, Google Scholar

**Sökstrategi (search strategy):** se sid 2 (see page 2) –

**Sökhistorik/Search history**

**Database:** Ovid MEDLINE(R) ALL (OvidSP)

**Date:** 2023‐09‐19

**No of results:** 93 refs

**Table:**

#	Searches	Results
1	exp Ultrasonography/	488,809
2	exp Ultrasonics/	26,105
3	(ultrason* or ultra‐son* or ultrasound* or ultra‐sound* or US‐guid*).ab,kf,ti.	466,965
4	1 or 2 or 3	741,618
5	exp Anaesthesia, Conduction/	71,856
6	exp Anesthetics, Local/	111,720
7	(((anesth* or anaesth*) adj5 (regional or conduction or local or locoregional or topical or infiltration* or epidural or peridural or extradural or spinal or ophthalmic or otic)) or ((nerve* or neurogenic* or neuro‐genic* or ganglion* or brachial or cervical or plexus* or autonomic or conduction or paracervical or quadratus lumborum or retrobulbar or retroocular or (transvers* adj abdomin* adj plane)) adj5 block*) or gangliopleg*).ab,kf,ti.	123,133
8	5 or 6 or 7	218,868
9	exp Artificial Intelligence/	179,436
10	(((artificial or machin* or comput* or automated) adj3 (intellig* or superintellig* or reason* or vision* or knowledge* or inferen*)) or (knowledge adj3 acquisition*) or AI‐guid* or AI‐assist* or AI‐aid* or AI‐augment* or AI‐base* or AI‐system* or ((comput* or algorithm*) adj3 heuristic*) or hyperheuristic* or hyper‐heuristic* or metaheuristic* or meta‐heuristic* or ((multicriteria or multi‐criteria or “multiple criteria” or multiobjective or multi‐objective or multiattribute or multi‐attribute) adj3 (decision* or optimi* or algorithm*)) or MOEA or (analytic* adj3 hierarch*) or (cognitive adj3 (comput* or robotic* or technol*)) or ((machine or transfer or deep or hierarchical or labeled data or supervised or semisupervised or unsupervised or network* or Bayesian or manifold*) adj3 learn*) or ((automat* or software or system* or technol* or pattern* or optical character*) adj3 recognit*) or (back* adj3 propagation*) or backpropagation* or ((classificat* or detect* or learn*) adj3 algorithm*) or classifier* or ((confusion or error or matching) adj3 matri*) or ((feature or edge) adj3 (detect* or extract* or learn* or rank* or select*)) or (vector* adj3 (machine* or support or classif* or network* or regression*)) or (expert adj3 (system or systems)) or (fuzz* adj3 (logic* or system*)) or hidden Markov or Markov state model* or iterative closest point* or knearest neighbo* or k nearest neighbo* or kernel* or qlearn* or q‐learn* or (temporal difference adj3 (algorithm* or learn*)) or ((multifactor* or multi‐factor*) adj3 dimension* adj3 reduction*) or (online analytic* adj3 process*) or OLAP or ((outlier or anomaly) adj3 detect*) or (radial bas* adj3 function*) or (general* adj3 regression adj3 network*) or random forest* or knowledge base or knowledge bases or ((biologic or biological or biomedical or gene) adj3 ontolog*) or (natural language adj3 (process* or system*)) or language process* or NLP or ((neural or deep or convolutional or feedforward or feed‐forward) adj3 network*) or memristor* or molecular dock* or perceptron* or (connect* adj3 (model* or network* or system*)) or autoencoder* or auto‐encoder* or ((companion or social or socially) adj3 (robot or robots)) or (sentiment adj3 (analys* or classif*)) or datamining or data‐mining or opinion mining* or (recursive adj3 (partitioning* or feature elimination*)) or (rough adj3 set*)).ab,kf,ti.	527,339
11	9 or 10	580,916
12	4 and 8 and 11	97
**13**	**limit 12 to (english or italian or swedish)**	**93**

**exp/**= term from the Medline controlled vocabulary, including terms found below this term in the MeSH hierarchy.

**ab,kf,ti**. = abstract, author keywords and title

**Adj3/adj5** = next to each other, in any order, up to 2/4 words in between

**adj** = next to each other, in that specific order

***** = truncation of word for alternate endings

**Database:** Embase 1974 to 2023 September 18

**Date:** 2023‐09‐19

**No of results:** 179 refs

**Table:**

#	Searches	Results
1	exp ultrasound/	227,546
2	exp echography/	1,003,728
3	(ultrason* or ultra‐son* or ultrasound* or ultra‐sound* or US‐guid*).ab,kf,ti.	691,529
4	1 or 2 or 3	1,386,944
5	exp regional anesthesia/	67,021
6	exp local anesthetic agent/	283,255
7	(((anesth* or anaesth*) adj5 (regional or conduction or local or locoregional or topical or infiltration* or epidural or peridural or extradural or spinal or ophthalmic or otic)) or ((nerve* or neurogenic* or neuro‐genic* or ganglion* or brachial or cervical or plexus* or autonomic or conduction or paracervical or quadratus lumborum or retrobulbar or retroocular or (transvers* adj abdomin* adj plane)) adj5 block*) or gangliopleg*).ab,kf,ti.	159,996
8	5 or 6 or 7	390,563
9	exp artificial intelligence/	86,055
10	exp machine learning/	416,450
11	exp computer vision/	3174
12	exp natural language processing/	10,590
13	(((artificial or machin* or comput* or automated) adj3 (intellig* or superintellig* or reason* or vision* or knowledge* or inferen*)) or (knowledge adj3 acquisition*) or AI‐guid* or AI‐assist* or AI‐aid* or AI‐augment* or AI‐base* or AI‐system* or ((comput* or algorithm*) adj3 heuristic*) or hyperheuristic* or hyper‐heuristic* or metaheuristic* or meta‐heuristic* or ((multicriteria or multi‐criteria or “multiple criteria” or multiobjective or multi‐objective or multiattribute or multi‐attribute) adj3 (decision* or optimi* or algorithm*)) or MOEA or (analytic* adj3 hierarch*) or (cognitive adj3 (comput* or robotic* or technol*)) or ((machine or transfer or deep or hierarchical or labeled data or supervised or semisupervised or unsupervised or network* or Bayesian or manifold*) adj3 learn*) or ((automat* or software or system* or technol* or pattern* or optical character*) adj3 recognit*) or (back* adj3 propagation*) or backpropagation* or ((classificat* or detect* or learn*) adj3 algorithm*) or classifier* or ((confusion or error or matching) adj3 matri*) or ((feature or edge) adj3 (detect* or extract* or learn* or rank* or select*)) or (vector* adj3 (machine* or support or classif* or network* or regression*)) or (expert adj3 (system or systems)) or (fuzz* adj3 (logic* or system*)) or hidden Markov or Markov state model* or iterative closest point* or knearest neighbo* or k nearest neighbo* or kernel* or qlearn* or q‐learn* or (temporal difference adj3 (algorithm* or learn*)) or ((multifactor* or multi‐factor*) adj3 dimension* adj3 reduction*) or (online analytic* adj3 process*) or OLAP or ((outlier or anomaly) adj3 detect*) or (radial bas* adj3 function*) or (general* adj3 regression adj3 network*) or random forest* or knowledge base or knowledge bases or ((biologic or biological or biomedical or gene) adj3 ontolog*) or (natural language adj3 (process* or system*)) or language process* or NLP or ((neural or deep or convolutional or feedforward or feed‐forward) adj3 network*) or memristor* or molecular dock* or perceptron* or (connect* adj3 (model* or network* or system*)) or autoencoder* or auto‐encoder* or ((companion or social or socially) adj3 (robot or robots)) or (sentiment adj3 (analys* or classif*)) or datamining or data‐mining or opinion mining* or (recursive adj3 (partitioning* or feature elimination*)) or (rough adj3 set*)).ab,kf,ti.	628,666
14	9 or 10 or 11 or 12 or 13	754,479
15	4 and 8 and 14	186
**16**	**limit 15 to (english or italian or swedish)**	**179**

**exp/**= term from the Embase controlled vocabulary, including terms found below this term in the Emtree hierarchy

**ab,kf,ti**. = abstract, author keywords and title

**adj3/adj5** = next to each other, in any order, up to 2/4 words in between

**adj** = next to each other, in that specific order

***** = truncation of word for alternate endings

**Database:** Cinahl

**Date:** 2023‐09‐19

**No of results:** 44 refs

**Table:**

#	Query	Results
**S13**	**S4 AND S8 AND S11** **Limiters ‐ Language: English, Italian, Swedish**	**44**
S12	S4 AND S8 AND S11	44
S11	S9 OR S10	113,206
S10	TI ((((artificial OR machin* OR comput* OR automated) N2 (intellig* OR superintellig* OR reason* OR vision* OR knowledge* OR inferen*)) OR (knowledge N2 acquisition*) OR AI‐guid* OR AI‐assist* OR AI‐aid* OR AI‐augment* OR AI‐base* OR AI‐system* OR ((comput* OR algorithm*) N2 heuristic*) OR hyperheuristic* OR hyper‐heuristic* OR metaheuristic* OR meta‐heuristic* OR ((multicriteria OR multi‐criteria OR “multiple criteria” OR multiobjective OR multi‐objective OR multiattribute OR multi‐attribute) N2 (decision* OR optimi* OR algorithm*)) OR MOEA OR (analytic* N2 hierarch*) OR (cognitive N2 (comput* OR robotic* OR technol*)) OR ((machine OR transfer OR deep OR hierarchical OR labeled data OR supervised OR semisupervised OR unsupervised OR network* OR Bayesian OR manifold*) N2 learn*) OR ((automat* OR software OR system* OR technol* OR pattern* OR optical character*) N2 recognit*) OR (back* N2 propagation*) OR backpropagation* OR ((classificat* OR detect* OR learn*) N2 algorithm*) OR classifier* OR ((confusion OR error OR matching) N2 matri*) OR ((feature OR edge) N2 (detect* OR extract* OR learn* OR rank* OR select*)) OR (vector* N2 (machine* OR support OR classif* OR network* OR regression*)) OR (expert N2 (system OR systems)) OR (fuzz* N2 (logic* OR system*)) OR hidden Markov OR Markov state model* OR iterative closest point* OR knearest neighbo* OR k nearest neighbo* OR kernel* OR qlearn* OR q‐learn* OR (temporal difference N2 (algorithm* or learn*)) OR ((multifactor* or multi‐factor*) N2 dimension* N2 reduction*) OR (online analytic* N2 process*) OR OLAP OR ((outlier OR anomaly) N2 detect*) OR (radial bas* N2 function*) OR (general* N2 regression N2 network*) OR random forest* OR knowledge base OR knowledge bases OR ((biologic OR biological OR biomedical OR gene) N2 ontolog*) OR (natural language N2 (process* OR system*)) OR language process* OR NLP OR ((neural OR deep OR convolutional OR feedforward OR feed‐forward) N2 network*) OR memristor* OR molecular dock* OR perceptron* OR (connect* N2 (model* OR network* OR system*)) OR autoencoder* OR auto‐encoder* OR robotic* OR telerobotic* OR ((companion OR social OR socially) N2 (robot OR robots)) OR ((sentiment) N2 (analys* OR classif*)) OR datamining OR data‐mining OR opinion mining* OR (recursive N2 (partitioning* OR feature elimination*)) OR (rough N2 set*))) OR AB (((artificial OR machin* OR comput* OR automated) N2 (intellig* OR superintellig* OR reason* OR vision* OR knowledge* OR inferen*)) OR (knowledge N2 acquisition*) OR AI‐guid* OR AI‐assist* OR AI‐aid* OR AI‐augment* OR AI‐base* OR AI‐system* OR ((comput* OR algorithm*) N2 heuristic*) OR hyperheuristic* OR hyper‐heuristic* OR metaheuristic* OR meta‐heuristic* OR ((multicriteria OR multi‐criteria OR “multiple criteria” OR multiobjective OR multi‐objective OR multiattribute OR multi‐attribute) N2 (decision* OR optimi* OR algorithm*)) OR MOEA OR (analytic* N2 hierarch*) OR (cognitive N2 (comput* OR robotic* OR technol*)) OR ((machine OR transfer OR deep OR hierarchical OR labeled data OR supervised OR semisupervised OR unsupervised OR network* OR Bayesian OR manifold*) N2 learn*) OR ((automat* OR software OR system* OR technol* OR pattern* OR optical character*) N2 recognit*) OR (back* N2 propagation*) OR backpropagation* OR ((classificat* OR detect* OR learn*) N2 algorithm*) OR classifier* OR ((confusion OR error OR matching) N2 matri*) OR ((feature OR edge) N2 (detect* OR extract* OR learn* OR rank* OR select*)) OR (vector* N2 (machine* OR support OR classif* OR network* OR regression*)) OR (expert N2 (system OR systems)) OR (fuzz* N2 (logic* OR system*)) OR hidden Markov OR Markov state model* OR iterative closest point* OR knearest neighbo* OR k nearest neighbo* OR kernel* OR qlearn* OR q‐learn* OR (temporal difference N2 (algorithm* or learn*)) OR ((multifactor* or multi‐factor*) N2 dimension* N2 reduction*) OR (online analytic* N2 process*) OR OLAP OR ((outlier OR anomaly) N2 detect*) OR (radial bas* N2 function*) OR (general* N2 regression N2 network*) OR random forest* OR knowledge base OR knowledge bases OR ((biologic OR biological OR biomedical OR gene) N2 ontolog*) OR (natural language N2 (process* OR system*)) OR language process* OR NLP OR ((neural OR deep OR convolutional OR feedforward OR feed‐forward) N2 network*) OR memristor* OR molecular dock* OR perceptron* OR (connect* N2 (model* OR network* OR system*)) OR autoencoder* OR auto‐encoder* OR robotic* OR telerobotic* OR ((companion OR social OR socially) N2 (robot OR robots)) OR ((sentiment) N2 (analys* OR classif*)) OR datamining OR data‐mining OR opinion mining* OR (recursive N2 (partitioning* OR feature elimination*)) OR (rough N2 set*))	101,208
S9	(MH “Artificial Intelligence+“)	30,794
S8	S5 OR S6 OR S7	45,382
S7	TI (((anesth* OR anaesth*) N4 (regional OR conduction OR local OR locoregional OR topical OR infiltration* OR epidural OR peridural OR extradural OR spinal OR ophthalmic OR otic)) OR ((nerve* OR neurogenic* OR neuro‐genic* OR ganglion* OR brachial OR cervical OR plexus* OR autonomic OR conduction OR paracervical OR quadratus lumborum OR retrobulbar OR retroocular OR (transvers* W0 abdomin* W0 plane)) N4 block*) OR gangliopleg*) OR AB (((anesth* OR anaesth*) N4 (regional OR conduction OR local OR locoregional OR topical OR infiltration* OR epidural OR peridural OR extradural OR spinal OR ophthalmic OR otic)) OR ((nerve* OR neurogenic* OR neuro‐genic* OR ganglion* OR brachial OR cervical OR plexus* OR autonomic OR conduction OR paracervical OR quadratus lumborum OR retrobulbar OR retroocular OR (transvers* W0 abdomin* W0 plane)) N4 block*) OR gangliopleg*)	26,401
S6	(MH “Anesthetics, Local+“)	19,529
S5	(MH “Anesthesia, Conduction+“)	21,234
S4	S1 OR S2 OR S3	173,426
S3	TI (ultrason* OR ultra‐son* OR ultrasound* OR ultra‐sound* OR US‐guid*) OR AB (ultrason* OR ultra‐son* OR ultrasound* OR ultra‐sound* OR US‐guid*)	100,780
S2	(MH “Ultrasonics+“)	2,018
S1	(MH “Ultrasonography+“)	115,968

**MH”“+** = term from the Cinahl controlled vocabulary, including terms found below this term in the hierarchy

**AB** = abstract

**TI** = title

**N2/N4** = next to each other, in any order, up to 2/4 words in between

**W0** = next to each other, in that specific order

***** = truncation of word for alternate endings

**Database:** The Cochrane Library

**Date:** 2023‐09‐19

**No of results:** 10 refs

*Cochrane reviews: 0*

*Cochrane protocols: 0*

*Trials: 10*

*Editorials: 0*

*Special collections: 0*

*Clinical answers: 0*

**Table:**

ID	Search	Hits
#1	MeSH descriptor: [Ultrasonography] explode all trees	17,620
#2	MeSH descriptor: [Ultrasonics] explode all trees	386
#3	(ultrason* OR (ultra NEXT son*) OR ultrasound* OR (ultra NEXT sound*) OR (US NEXT guid*)):ti,ab,kw (Word variations have been searched)	56,036
#4	#1 OR #2 OR #3	61,711
#5	MeSH descriptor: [Anesthesia, Conduction] explode all trees	12,194
#6	MeSH descriptor: [Anesthetics, Local] explode all trees	9871
#7	(((anesth* OR anaesth*) NEAR/4 (regional OR conduction OR local OR locoregional OR topical OR infiltration* OR epidural OR peridural OR extradural OR spinal OR ophthalmic OR otic)) OR ((nerve* OR neurogenic* OR (neuro NEXT genic*) OR ganglion* OR brachial OR cervical OR plexus* OR autonomic OR conduction OR paracervical OR quadratus lumborum OR retrobulbar OR retroocular OR (transvers* NEXT abdomin* NEXT plane)) NEAR/4 block*) OR gangliopleg*):ti,ab,kw (Word variations have been searched)	51,887
#8	#5 OR #6 OR #7	51,925
#9	MeSH descriptor: [Artificial Intelligence] explode all trees	2929
#10	(((artificial OR machin* OR comput* OR automated) NEAR/2 (intellig* OR superintellig* OR reason* OR vision* OR knowledge* OR inferen*)) OR (knowledge NEAR/2 acquisition*) OR (AI NEXT guid*) OR (AI NEXT assist*) OR (AI NEXT aid*) OR (AI NEXT augment*) OR (AI NEXT base*) OR (AI NEXT system*) OR ((comput* OR algorithm*) NEAR/2 heuristic*) OR hyperheuristic* OR (hyper NEXT heuristic*) OR metaheuristic* OR (meta NEXT heuristic*) OR ((multicriteria OR (multi NEXT criteria) OR (multiple NEXT criteria) OR multiobjective OR (multi NEXT objective) OR multiattribute OR (multi NEXT attribute)) NEAR/2 (decision* OR optimi* OR algorithm*)) OR MOEA OR (analytic* NEAR/2 hierarch*) OR (cognitive NEAR/2 (comput* OR robotic* OR technol*)) OR ((machine OR transfer OR deep OR hierarchical OR (labeled NEXT data) OR supervised OR semisupervised OR unsupervised OR network* OR Bayesian OR manifold*) NEAR/2 learn*) OR ((automat* OR software OR system* OR technol* OR pattern* OR (optical NEXT character*)) NEAR/2 recognit*) OR (back* NEAR/2 propagation*) OR backpropagation* OR ((classificat* OR detect* OR learn*) NEAR/2 algorithm*) OR classifier* OR ((confusion OR error OR matching) NEAR/2 matri*) OR ((feature OR edge) NEAR/2 (detect* OR extract* OR learn* OR rank* OR select*)) OR (vector* NEAR/2 (machine* OR support OR classif* OR network* OR regression*)) OR (expert NEAR/2 (system OR systems)) OR (fuzz* NEAR/2 (logic* OR system*)) OR (hidden NEXT Markov) OR (Markov NEXT state NEXT model*) OR (iterative NEXT closest NEXT point*) OR (knearest NEXT neighbo*) OR (k NEXT nearest NEXT neighbo*) OR kernel* OR qlearn* OR (q NEXT learn*) OR ((temporal NEXT difference) NEAR/2 (algorithm* or learn*)) OR ((multifactor* or (multi NEXT factor*)) NEAR/2 dimension* NEAR/2 reduction*) OR ((online NEXT analytic*) NEAR/2 process*) OR OLAP OR ((outlier OR anomaly) NEAR/2 detect*) OR ((radial NEXT bas*) NEAR/2 function*) OR (general* NEAR/2 regression NEAR/2 network*) OR (random NEXT forest*) OR (knowledge NEXT base) OR (knowledge NEXT bases) OR ((biologic OR biological OR biomedical OR gene) NEAR/2 ontolog*) OR ((natural NEXT language) NEAR/2 (process* OR system*)) OR (language NEXT process*) OR NLP OR ((neural OR deep OR convolutional OR feedforward OR (feed NEXT forward)) NEAR/2 network*) OR memristor* OR (molecular NEXT dock*) OR perceptron* OR (connect* NEAR/2 (model* OR network* OR system*)) OR autoencoder* OR (auto NEXT encoder*) OR ((companion OR social OR socially) NEAR/2 (robot OR robots)) OR ((sentiment) NEAR/2 (analys* OR classif*)) OR datamining OR (data NEXT mining) OR (opinion NEXT mining*) OR (recursive NEAR/2 (partitioning* OR (feature NEXT elimination*))) OR (rough NEAR/2 set*)):ti (Word variations have been searched)	4538
#11	#9 OR #10	6332
#12	#4 AND #8 AND #11	10

**MeSH descriptor: [] explode all trees** = term from the MeSH controlled vocabulary, including terms found below this term in the MeSH hierarchy

**ab** = abstract

**kw** = author keywords

**ti** = title

**NEAR/2/NEAR/4 =** next to each other, in any order, up to 2/4 words in between

**NEXT =** next to each other, in that specific order

***** = truncation of word for alternate endings

**Source:** Google Scholar (using Publish or Perish software)

**Date:** 2023‐09‐20

**No of exported results:** 100 refs

**Table:**

Sökord/Begränsningar	Antal exporterade referenser	Kommentarer ang urval av referenser
(ultrasound OR ultrasonography OR ultrasonics) AND (regional anesthesia OR conduction anesthesia OR local anesthesia OR nerve block) AND (artificial intelligence OR AI OR machine learning OR ML OR deep learning) *Sökte i fältet Keywords i Publish or Perish*	100	Jag exporterade de första hundra träffarna till EndNote. Därefter minskade relevansen markant.

## APPENDIX 2

See Table A2.

**Table A2:** Artificial intelligence (AI) algorithm and corresponding characteristics.

Algorithm	Function	Description	Open source
Particle filter (PF)	Feature tracking	PF is a mathematical algorithm used to find approximate solutions for filtering problems in nonlinear state‐space systems.	Open‐source implementations are available.
Mean shift (MS)	Feature tracking	Nonparametric feature‐space mathematical analysis technique used to locate the maxima (modes) of a density function. It is often referred to as a mode‐seeking algorithm.	Open‐source implementations are available.
Kanade–Lucas–Tomasi (KLT)	Feature tracking	It focuses on tracking feature points across consecutive frames in videos or image sequences.	Open‐source implementations are available.
Efficient convolution operators (ECO)	Feature tracking	Developed to address challenges faced by traditional methods of object tracking in terms of computational complexity and overfitting and aims to improve both tracking speed and performance.	Open‐source implementations are available.
Hedged deep tracking (HDT)	Feature tracking	Innovative CNN‐based tracking algorithm designed for visual tracking and combines several trackers into a stronger one to improve tracking accuracy by leveraging features from multiple layers.	Open‐source implementations are available.
Structure‐aware network (SANet)	Feature tracking	Most existing CNN‐based trackers treat tracking as a classification problem, and they can be sensitive to similar distractors due to their focus on interclass classification. SANet instead incorporates a recurrent neural network (RNN) to model the object's structure. By combining RNN with CNN, SANet improves robustness to similar distractors.	Open‐source implementations are available.
Continuous convolution operators tracker (C‐COT)	Feature tracking	Is an advanced visual tracking algorithm that introduces a novel formulation for training continuous convolution filters. It employs an implicit interpolation model to pose the learning problem in the continuous spatial domain. The key innovation is the efficient integration of multi‐resolution deep feature maps.	Open‐source implementations are available.
Joint adaptive median binary pattern (JAMBP)	Feature extracting	Texture descriptor designed for texture classification and recognition.	Open‐source implementations are available.
Histograms of Oriented Gradients (HOG)	Feature extracting	Powerful feature descriptor used in computer vision and image processing for object detection.	Open‐source implementations are available.
AlexNet	Convolutional neural network (CNN)	AlexNet is a pioneering CNN architecture that has introduced several innovations including depth, rectified linear unit activation function that accelerates training speed, and it incorporates dropout layers during training to prevent overfitting.	Open‐source implementations are available.
GoogLeNet	CNN	It is a CNN architecture designed to address the limitations of previous architectures like AlexNet and ZF‐Net. Its primary goal was to create a deeper network while maintaining computational efficiency.	Open‐source implementations are available.
ResNet‐50	CNN	Residual Network, with 50 layers, is a powerful CNN architecture. It is widely used for image classification tasks.	Open‐source implementations are available.
SqueezeNet	CNN	It is as CNN architecture. It was designed to achieve competitive accuracy while significantly reducing the number of parameters.	Open‐source implementations are available.
ScanNav	AI‐assistance software	ScanNav Anatomy Peripheral Nerve Block (PNB) is an advanced real‐time AI‐assistance software designed specifically for UGRA. It enhances accuracy by providing a colour overlay of key sono‐anatomical structures during live ultrasound scans.	Not open source
SegNet Model	CNN	Is a deep fully convolutional neural network architecture designed for semantic pixel‐wise segmentation. It consists of an encoder network, a corresponding decoder network and a pixel‐wise classification layer.	Not open source
Nerveblox	AI‐assistance software	Is an advanced AI solution for ultrasound‐guided peripheral nerve blocks. It assists anaesthesiologists by capturing real‐time ultrasound images and automatically labelling key anatomical landmarks.	Not open source
